# Diagnostic performance of isolated-check visual evoked potential versus retinal ganglion cell-inner plexiform layer analysis in early primary open-angle glaucoma

**DOI:** 10.1186/s12886-017-0472-9

**Published:** 2017-05-22

**Authors:** XiangWu Chen, YingXi Zhao

**Affiliations:** Department of Out Patient Service, The Eye Hospital of Wenzhou Medical University, Postal code: 325000, Wenzhou, Zhejiang Province China

**Keywords:** Isolated-check visual evoked potential, Optical coherence tomography, Signal-to-noise ratios, Glaucomatous optic neuropathy

## Abstract

**Background:**

The purpose of this study was to compare the diagnostic performance of isolated-check visual evoked potential (icVEP) with that of retinal ganglion cell-inner plexiform layer (GCILP) analysis using optical coherence tomography (OCT).

**Methods:**

A total of 45 patients were enrolled: 25 patients with open-angle glaucoma and 20 healthy patients. All patients underwent a complete ophthalmological examination. Moreover, the OCT examination was used to analyze the structures of the GCIPL. The icVEP technique was used to detect the transmission function of the magnocellular pathway, which is mainly managed by the retinal ganglion cells. The quantitative and qualitative comparisons between the diagnostic power of GCIPL analysis and that of icVEP were performed. The areas under the receiver operating characteristic curves (AUC) of GCIPL analysis and icVEP were compared using the Clarke-Pearson method. The sensitivity and specificity of the two techniques were analyzed and compared using the McNemar test.

**Results:**

With the quantitative comparison, the AUC of icVEP (AUC = 0.892) was higher than that of GCIPL analysis (AUC = 0.814). However, there was no statistical significance between the AUCs of icVEP and GCIPL (*P* > 0.05). With the qualitative comparison, the sensitivity of icVEP was 80%, and its specificity was 90%. The sensitivity of GCIPL analysis was 72%, and its specificity was 85%. There was no significant difference between the sensitivitiesor specificities of icVEP and GCIPL analysis (*P* > 0.05). Moreover, 30 (66.67%) eyeshad similar resultsbetween icVEP and GCIPL analysis, and 15 (33.33%) eyes had different results (7 eyes had abnormal results with GCIPL analysisbut normal results with icVEP, and8 eyes had normal results with GCIPL analysisbut abnormal results with icVEP).

**Conclusions:**

The diagnostic power of icVEP was close to that of GCIPL analysis whether the comparison was based on the qualitative or quantitative data.

## Background

Primary open angle glaucoma is a progressive optic neuropathy that is usually caused by high pressure inside the eye and characterized by gradual degeneration of the retinal ganglion cells (RGC) [1]. The most widely used method of functional assessment for diagnosing glaucoma is visual field analysis, which relies on behavioral responses to detect functional deficits [2]. However, by the time these visual field deficits are detected, there is a significant reduction in the RGC population [3]. Therefore, examination by visual field analysis is not adequate for the early diagnosis of glaucoma.

Two advanced techniques have been introduced to the field to assess the RGC abnormalities associated with glaucoma. One is optical coherence tomography (OCT), which facilitates the quantitative and qualitative analyses of the structures of the retinal ganglion cell-inner plexiform layer (GCIPL) [4, 5]. Another is isolated-check visual evoked potential (icVEP), which is designed to detect the transmission function of the magnocellular pathway, a neural pathway that is mainly managed by the RGCs [6]. The GCIPL analysis is used to detect early glaucoma by assessing the structural changes of macular RGCs [7–9] whereas the icVEP is used to diagnose early glaucoma by identifying the functional abnormalities of macular RGCs. Although GCIPL analysis and icVEP techniques target the same neuroretinal cells, the detection mechanisms of these two techniques are entirely different. This study investigated the above tests to determine which one performs better in the diagnosis of early glaucoma.

Therefore, the primary purpose of the current study is to compare the diagnostic power of icVEP and GCIPL analysis quantitatively and qualitatively at the RGC level in eyes with early primary open angle glaucoma.

## Methods

### Patients

Participants were recruited from the Eye Hospital of Wenzhou Medical University. All patients signed informed consent forms prior to participation. The Tenets of the Declaration of Helsinki were followed. This study was approved by the Ethics Committee of the Eye Hospital of Wenzhou Medical University. All participants underwent the necessary ophthalmic examinations, including visual acuity, slit-lamp biomicroscopy, refraction, gonioscopy, Goldmann applanation tonometry, visual field analysis, icVEP, and OCT. The visual field analysis was performed using the Humphrey Field Analyzer (HFA) II (model 750; Carl Zeiss Meditec, Inc.). To reduce learning effects, all participants took at least two HFA tests. The results of visual field analysis were considered reliable when fixation losses were <20% and false positive and negative errors were <33% [10].

The eyes were classified into the early glaucoma group or the control group. The inclusion criteria for the early glaucoma group were as follows: eyes with open anterior chamber angle and glaucomatous optic neuropathy (cup-disc ratio ≥ 0.6 or vertical cup/disc diameter ratio asymmetry ≥0.2), central corneal thickness (CCT) adjusted for untreated intraocular pressure > 21 mmHg, visual field defects on standard automated perimetry (SAP) (glaucoma hemifield test results that were outside normal limits; pattern standard deviation (PSD) with *p* value <5%; or cluster of three or more non-edge points on the pattern deviation plot in a single hemi-field with *p* values <5%, one of which must have a *p* value <1% [10]), and mean deviation of the visual field at ≤6 dB. The inclusion criteria for the control group were as follows: no family history of glaucoma; intraocular pressure (IOP) <21 mmHg; a normal visual field; a central thickness of cornea >500 mm; normal optic nerve head; normal retinal nerve fibre layer.

Patients were excluded from this study if they met one of the following conditions: best corrected visual acuity <20/30; spectacle refraction > ±5.00D sphere or > ± 2.00D cylinder; pupil diameter < 2.5 mm; diseases that could lead to visual field loss or affect macular thickness; former ocular surgery (except for uncomplicated cataract surgery); or neurological diseases that may influence the icVEP results (e.g., amblyopia, ischemic optic neuropathy, etc.).

### Isolated-check visual evoked potential

The icVEP was performed using the Neucodia visual electrophysiological diagnostic system (MKWH AMD, Huzhou Medconova Medical Technology, Inc.). At the beginning of icVEP testing, electrodes were connected to the scalp of patients using an electrolytic paste. Patients were instructed to listen for an auditory cue and to stare at a cross in the center of a computer screen, which was going to display a specific visual pattern. Elicited by this special picture, the subjects’ cortical response was recorded by the Neucodia system, which presented the outcome as an electroencephalogram (EEG). This process totally took 2 s to finish (1 s each for EEG testing and recording). Next, the EEG was transformed into the fundamental frequency component (FFC), which is an important intermediate parameter in the examination. After eight separate runs, the instrument calculated the mean FFC and the radius of the 95% confidence circle [6].

**Fig. 1:**
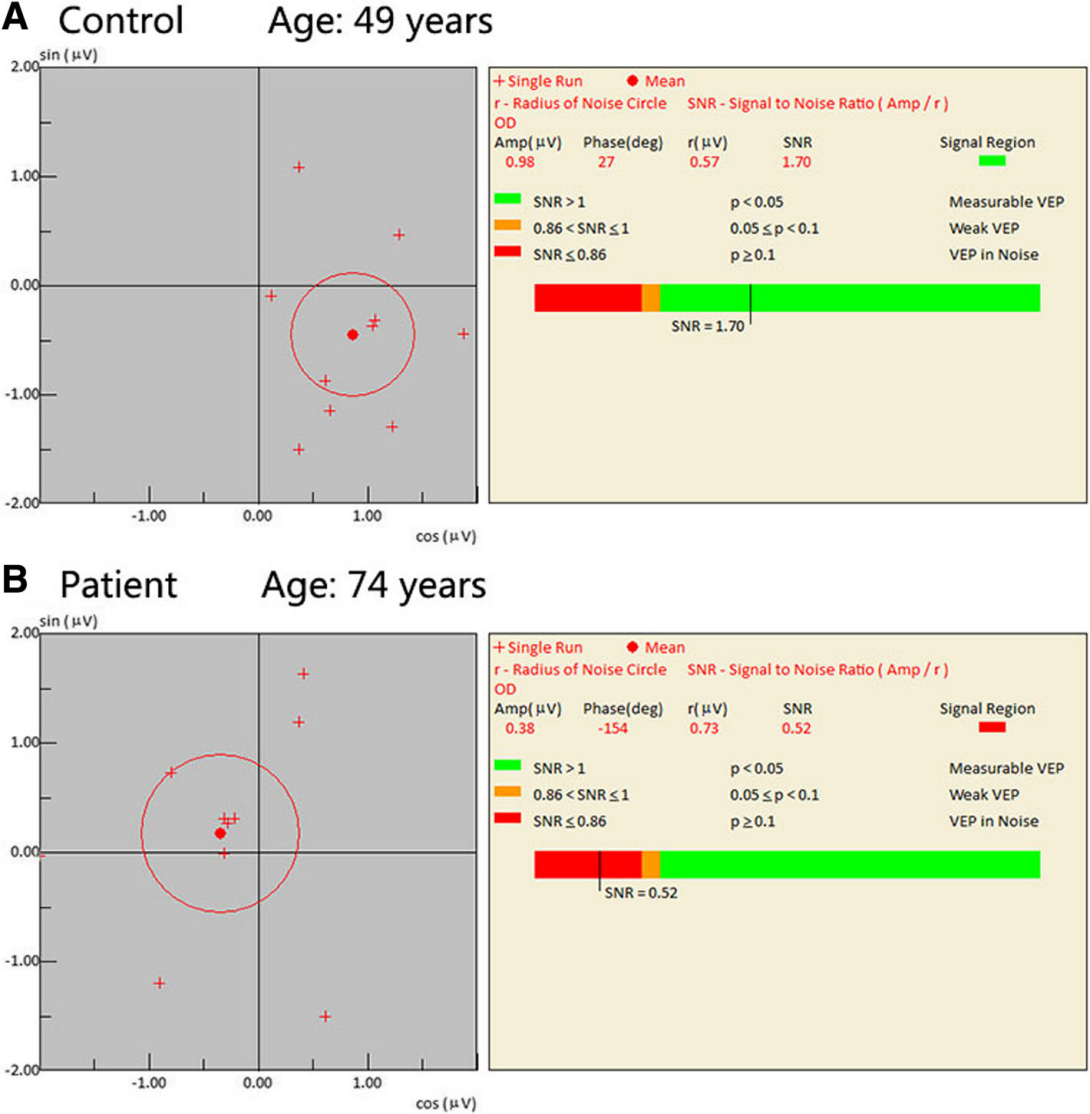
Eight separate fundamental frequency components of the icVEP under the 15% bright-check condition. **a** normal; **b** abnormal

The signal-to-noise ratio (SNR) was defined as the ratio of the mean amplitude of FFC to the radius of the 95% confidence circle. The SNR was the final number that was used to determine the presence of glaucomatous damage. SNR ≤1.0 was defined as abnormal, whereas SNR>1.0 was defined as normal [6, 11] (Fig. 1). In this study, 15% positive-contrast (bright) condition pattern was used to differentiate between healthy participants and glaucoma patients.

**Fig. 2:**
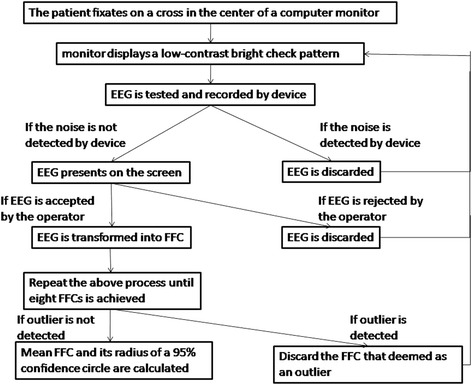
The procedure for the icVEP test

To obtain reliable SNR, three methods were used in this test: automated noise detection, automated outlier analysis, and operator verification. The techniques for noise detection and outlier analysis were incorporated in the program of Neucodia system. If noise was detected by the device during the testing process, the elicited EEG was discarded and the program would prompt the operator to repeat the run. If noise was not detected, the EEG would be displayed on the screen and either be accepted or rejected by the operator depending on whether proper fixation was maintained during the test. After the eight FFCs were completed, outlier analysis was performed to identify whether one of the FFCs was an outlier relative to the other seven runs based on a statistical criterion. If a run was identified as an outlier, the device will discard it and prompt the operator to repeat the test until eight qualified outcomes were collected. The process is shown in Fig. 2.

### Optical coherence tomography

OCT examination was performed using the Cirrus HD-OCT (Carl Zeiss Meditec, Dublin, California) on the same day as icVEP testing. The operators who performed the OCT examination were different from those who performed the icVEP testing. Three OCT volume scans centered on the macula were performed for GCIPL analysis. The signal strength of the OCT was analyzed. Out of the three scans, only the one with the best signal strength was selected for GCIPL analysis.

The minimal (lowest GCIPL thickness), average, and sectoral (inferotemporal, superior, inferior, inferonasal, superonasal, superotemporal) thicknesses of the GCIPL were measured in an elliptical annulus around the fovea. Some studies have reported that the minimal GCIPL thickness assessment was better than the other two GCIPL analyses for diagnosing glaucoma [4, 5]. Thus, the minimal GCIPL analysis was selected as the only indicator to assess the structural abnormalities in this study.

**Fig. 3:**
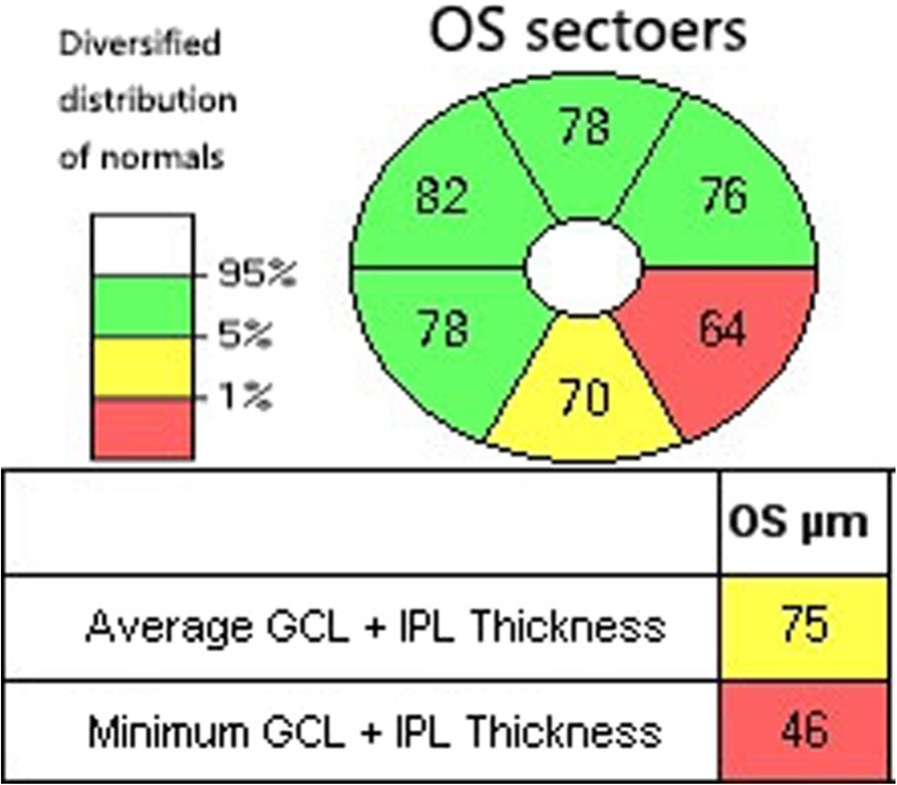
Optical coherence tomography images of the left eye of a patient with early glaucoma. In this case, the GCIPL sectors in the temporal inferior area was outside of normal limits (*red*) and the inferior area was borderline (*yellow*). The minimum GCIPL was outside of normal limits (*red*), and the average GCIPL was borderline (*yellow*)

Furthermore, Cirrus software automatically organized the GCIPL values into three categories: within normal limits (green), borderline (yellow), and outside of normal limits (red) (Fig. 3). To maintain higher specificity, the eyes classified as “borderline (yellow)” were included in the category of “within normal limits” in this study [12, 13].

### Statistical analysis

Continuous data were presented as mean ± standard deviation (SD). The receiver operating characteristic (ROC) curves were used to determine the discriminatory capabilities of the tests between glaucomatous and healthy eyes. The area under the ROC curves (AUC) were evaluated for minimal GCIPL thickness and SNR values. These quantitative values of the AUCs were compared using the Clarke-Pearson method for paired data.

The sensitivity was defined as the percentage of glaucomatous eyes that were abnormal in the structural or functional test. The specificity was defined as the percentage of control eyes that were normal in the structural or functional test. The comparison between sensitivity (or specificity) of icVEP and that of OCT was performed using the McNemar test. The level of significance was *p* < 0.05.

## Results

### Demographic characteristics of the study participants

A total of45 eyes from 45 patients were enrolled in this study: 25 eyes were assigned to the early glaucoma group and 20 eyes were assigned to the control group. The range of ages was from 44 to 77 years (60.96 ± 9.56 years). The demographic characteristics of the study participants are summarized in Table 1.

**Table 1:** Demographic characteristics of the study participants

Variable	Glaucoma group	Control group	Total	*P*-value
Number of cases	25	20	45	
Age in years (mean ± SD)	59.24 ± 9.88	61.55 ± 10.05	60.96 ± 9.56	0.944
Sex (male/female)	17/8	12/8	29/16	0.577
Spherical equivalents in diopters (mean ± SD)	0.4 ± 3.22	−0.26 ± 2.79	−0.09 ± 3.01	0.26

### Quantitative comparisons between icVEP and OCT

**Fig. 4:**
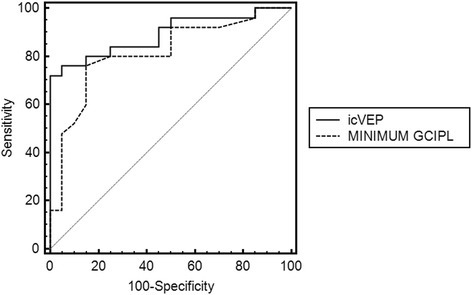
The ROC curves of icVEP and minimum GCIPL

Fig. 4 shows the ROC curves of icVEP and minimum GCIPL. Table 2 shows the AUCs, standard error, 95% confidence interval, and significance of these two techniques. The AUC of icVEP (AUC = 0.892) was higher than that of minimum GCIPL (AUC = 0.814);however, the difference was not statistically significant (z = 0.93, *p* = 0.356).

**Table 2:** AUC, standard error, 95% confidence interval and significance of icVEP and minimum GCIPL

	AUC	Standard error	95% CI	*P* value
icVEP	0.892	0.0488	0.763 to 0.965	<0.0001
Minimum GCIPL	0.814	0.0664	0.670 to 0.914	<0.0001

### Qualitative comparisons between icVEP and OCT

The classification of patients according to test results is listed in Table 3. In the early glaucoma group, 20 eyes on icVEP and 18 eyes on OCT were abnormal. In the control group, 18 eyes on icVEP and 17 eyes on OCT were normal. Thus, the sensitivity of icVEP was 80%, and its specificity was 90%. The sensitivity of OCT was 72%, and its specificity was 85%. The McNemar test revealed that there was no statistically significant difference between the sensitivity (or specificity) of icVEP and minimum GCIPL (sensitivity, *p* = 0.75; specificity, *p* = 1.00). In total, 30 (66.67%) eyes had similar results between icVEP and GCIPL analysis, and 15 (33.33%) eyes had different results (7 eyes had abnormal results with GCIPL analysis but normal results with icVEP, and 8 eyes had normal results with GCIPL analysis but abnormal results with icVEP).

**Table 3:** Contingency table of groupsversus test results

		Early glaucoma group	Control group
IcVEP	+	20	2
	−	5	18
Minimum GCIPL	+	18	3
	−	7	17

“+” = abnormality; “-” = normality

## Discussion

In this study, the quantitative comparison between the diagnostic performance of icVEP and that of OCT was performed using the ROC curve analyses. We discovered that although the absolute AUC value of SNR (derived from icVEP) was relatively higher than that of minimum GCIPL (derived from OCT) in this study, the statistical difference between these two AUC values was not significant. This result suggested that the diagnostic power of icVEP was similar to that of minimum GCIPL in the detection of early glaucoma when the comparison was based on quantitative data. However, the relatively higher AUC value of icVEP in this paper may not be purely by chance.

Several studies have supported that the AUC value of icVEP would be higher than that of OCT in the diagnosis of early glaucoma. First, studies have found that the individual GCIPL thickness varies with several factors, such as individual variation, age, and race [14]. This variation in GCIPL thickness presented a bias that would lower the diagnostic power of this technique in the diagnosis of glaucoma. Second, it was discovered that the RGCs with large diameter axons were preferentially damaged in early glaucoma [15, 16], and icVEP specialized in detecting the transmission function of the magnocellular pathway, which is mainly managed by the RGCs with large-diameter axons [17, 18]. However, the GCIPL analysis in OCT measured the retinal thicknesses from the ganglion cell layer to the inner plexiform layer [14], regardless of whether the RGCs with large diameter axons were included or not. This may also lower the diagnostic performance of GCIPL analysis in the detection of early glaucoma. Finally, it must be considered that in patients with glaucoma, there are two sources of functional impairment: one at the retinal level and another at the post-retinal level (such as at the lateral geniculate nucleus level [9]). icVEP examined the functional integrity of central vision at all levels of the visual pathway including the retina, optic nerve, optic radiations, and occipital cortex. However, OCT only detected the morphological abnormalities at the retinal level. These indicate that icVEP was more likely to identify glaucoma damage than OCT in the whole visual pathway. To sum up, the above three points strongly supported that icVEP would perform better than the minimum GCIPL analysis in the detection of early glaucoma. However, there was no statistically significant difference between the AUC values of icVEP and minimum GCIPL in this study. Since sample size plays an important role in statistical analysis, further research is needed to determine whether the statistical result would change when a new study with a larger sample size is performed.

Qualitative comparisons between icVEP and minimum GCIPL were also performed in this study. In contrast to the icVEP test, the qualitative assessment of GCIPL analysis was not simply cutoff values of the macular thicknesses but were attributes given by the device, which took into account more relevant information (such as age and race) to assign a result to a given category. Therefore, the information obtained with the qualitative assessment of GICPL analysis was more robust than that of the quantitative assessment of GCIPL analysis. In this study, the sensitivity and specificity of these two examinations were calculated from the qualitative data. We discovered that there were no statistically significant differences between the sensitivities or specificities of these two techniques (*p* > 0.05). This revealed that the overall performance of icVEP was close to that of the minimum GCIPL when the comparison was based on the qualitative data. However, the disagreements between icVEP and minimum GCIPL were 15 (33.33%) eyes, suggesting that icVEP detected some real abnormalities that minimum GCIPL did not and vice versa. This may be partially attributable to the variability of both techniques, but it is also possible that the functional abnormalities in early glaucoma did not manifest at the same time as the structural abnormalities. This viewpoint is supported by the findings of Higginbotham et al., who reported that structural progression accounted for nearly 60% of all conversions in early stage glaucoma, functional changes were responsible for 40%, and both structural and functional abnormalities accounted for less than 15% [19]. Hence, the detection of structural or functional abnormalities alone would lead to missed diagnosis of early glaucoma. Therefore, a combined evaluation of structural and functional changes may improve the detection of early glaucoma.

The present study has several limitations. The patients assigned to the glaucoma group all had eyes with early stage glaucoma (the mean deviation of visual field was ≤ 6 dB). Thus, the diagnostic performance of these two techniques would change if the patients with more serious stage glaucoma were enrolled in this study. Another limitation of this study was that the small sample size in the current study does not provide strong evidence for the results, which were based on the quantitative and qualitative comparisons between icVEP and OCT. Finally, peripheral visual function is known to be preferentially affected in the mild stage of glaucoma [20]. However, icVEP was designed to detect the central visual abnormalities of glaucoma. Further studies are needed to determine which examination is better in the diagnosis of early glaucoma: icVEP or techniques that were designed to identify the peripheral visual function, such as standard automated perimetry and multifocal visual evoked potential.

## Conclusions

In conclusion, the diagnostic power of icVEP was close to that of GCIPL analysis whether the comparison was based on the qualitative or quantitative data in this study. Several studies have revealed that a combination of structural and functional assessments may improve the detection of early glaucoma [21]. Thus, both OCT and icVEP should be used in the future to search for an optimal tool in the detection of early glaucoma.

## Abbreviations

AUC: Areas under the curve
CCT: Central corneal thickness
EEG: Electroencephalogram
FFC: Fundamental frequency component
GCILP: Retinal ganglion cell-inner plexiform layer
icVEP: Isolated-check visual evoked potential
IOP: Intraocular pressure
OCT: Optical coherence tomography
RGC: Retinal ganglion cells
ROC: Receiver operating characteristic
SAP: Standard automated perimetry
SD: Standard deviation
SNR: Signal-to-noise ratio
